# Capsaicin 8% Patch Treatment in Non-Freezing Cold Injury: Evidence for Pain Relief and Nerve Regeneration

**DOI:** 10.3389/fneur.2021.722875

**Published:** 2021-08-19

**Authors:** Praveen Anand, Rosario Privitera, Philippe Donatien, V. Peter Misra, David R. Woods

**Affiliations:** ^1^Department of Neurology, Imperial College London, Hammersmith Hospital, London, United Kingdom; ^2^Research and Clinical Innovation, Royal Centre for Defence Medicine, Birmingham, United Kingdom; ^3^Leeds Beckett University, Leeds, United Kingdom

**Keywords:** capsaicin 8% patch, non-freezing cold injury, neuropathic pain, clinical trial, skin biopsy

## Abstract

**Introduction:** Neuropathic pain associated with Non-freezing Cold Injury (NFCI) is a major burden to military service personnel. A key feature of NFCI is reduction of the intra-epidermal nerve fibre density in skin biopsies, in keeping with painful neuropathy. Current oral treatments are generally ineffective and have undesirable side effects. Capsaicin 8% patch (Qutenza) has been shown to be well-tolerated and effective for reducing neuropathic pain, for up to 3 months after a single 30-minute application.

**Methods:** In this single-centre open label study, 16 military participants with NFCI (mean duration 49 months) received 30-minute Capsaicin 8% patch treatment to the feet and distal calf. Pain symptoms were assessed using a pain diary (with the 11-point Numerical Pain Rating Scale, NPRS) and questionnaires, the investigations included skin biopsies, performed before and three months after treatment.

**Results:** Participants showed significant decrease in spontaneous pain (mean NPRS: −1.1, 95% CI: 0.37 to 1.90; *p* = 0.006), and cold-evoked pain (−1.2, 95% CI: 0.40 to 2.04; *p* = 0.006). The time-course of pain relief over 3 months was similar to other painful neuropathies. Patient Global Impression of Change showed improvement (*p* = 0.0001).

Skin punch biopsies performed 3 months after the patch application showed significant increase of nerve fibres with structural marker PGP9.5 (intra-epidermal nerve fibres [IENFs], *p* < 0.0001; sub-epidermal nerve fibres [SENFs]; *p* =< 0.0001), and of regenerating nerve fibres with their selective marker GAP43 (*p* = 0.0001). The increase of IENFs correlated with reduction of spontaneous (*p* = 0.027) and cold-evoked pain (*p* = 0.019).

**Conclusions:** Capsaicin 8% patch provides an exciting new prospect for treatment of NFCI, with regeneration and restoration of nerve fibres, for the first time, in addition to pain relief.

## Introduction

Cold temperatures may lead to different clinical injuries, depending on the rapidity, severity, and duration of the cold exposure. The condition now termed Non-freezing cold injury (NFCI) was previously called Trench Foot, and it has been known since World Wars I and II to be a vaso-neuropathy (1–3). NFCI and the associated chronic neuropathic pain are a particular burden to military personnel (3–9).

An underlying small fibre neuropathy and neuro-vascular changes have been demonstrated recently in skin biopsies, which may account for the chronic pain and persistent cold hypersensitivity (3). Currently prescribed oral treatments for neuropathic pain generally have limited efficacy, with multiple side effects. Further, there are no treatments that can cure or even modify the underlying condition. NFCI may lead to a downgrading of functional status, and in many cases early discharge from the armed services.

An effective treatment, licenced in the UK/EU for neuropathic pain, is the Capsaicin 8% patch (“Qutenza 179 mg cutaneous patch”), which may relieve pain for up to 3 months after a single 30-minute application (10–12). This treatment is localised to skin, and not associated with significant systemic absorption or generalised side effects. The active ingredient capsaicin is the substance in chilli peppers that gives them their hot pungency (10).

A key feature of NFCI with neuropathic pain is a reduction in intra-epidermal nerve fibre density in the skin, with dysfunction of the residual small sensory nerve fibres, as in many other causes of painful neuropathy. Thus, neuropathic pain associated with NFCI can be treated with the Capsaicin 8% patch, as licenced. The Capsaicin 8% patch has been shown by us in another chronic neuropathic condition (chemotherapy-induced painful neuropathy) to both relieve pain long term, and ameliorate the underlying pathological nerve damage, with regeneration and restoration of sensory nerve fibres (13).

In this study, we have investigated the effect of Capsaicin 8% patch treatment in subjects with NFCI related painful neuropathy, as licenced (i.e. 3-monthly, if required). Our aim was to relate any pain relief to sensory assessments and skin biopsy findings. These tests are used routinely for diagnosing and monitoring patients with small fibre neuropathy caused by a range of medical conditions. Our observations would potentially advance the understanding and management of NFCI.

## Materials and Methods

### Study Design

This was a single-centre longitudinal open label study on the effects of Capsaicin 8% patch treatment as licenced, to be conducted in 20 participants with NFCI related to military duties. Study approval was obtained from the Ministry of Defence Research Ethics Committee (Ethics reference number: 890/MoDREC/18).

### Participants

Twenty subjects consented to take part in the study. Data are reported here for the 16 participants who completed the follow-up visit at 3 months after single 30-minute application of Capsaicin 8% patch. Four participants withdrew from the study for personal reasons, unrelated to the study itself. Participants in the study received Capsaicin 8% patch application to both feet up to the distal calf. They continued to take their other medications (but not topical treatments) before and during the study.

Participant demographics and characteristics (*n* = 16) are recorded in Table 1.

**Table 1:** Participant demographics and characteristics.

**Parameters**	
Number of participants	*n* = 16
**Age, Years**	
Mean ± SD	36 ± 7.4
Range	25–56
**Sex, ***n*** (%)**	
Female	0 (0%)
Male	16 (100%)
**Duration of Non-freezing cold injury, Mean, Months (Range; SD)**	
	49 (10–120; 33)
**Ethnicity, ***n*** (%)**	
African/Caribbean	11 (69%)
Caucasian	4 (25%)
Other Ethnic origin	1 (6%)
**Exposure history, ***n*** (%)**	
Cold wet environment	7 (44%)
Cold windy environment	2 (13%)
Cold dry environment	7 (44%)
**Duration of exposure to cold environment before onset of symptoms, ***n*** (%)**	
<1h	2 (13%)
<24 h	5 (31%)
<1 week	2 (13%)
1 week	3 (19%)
>1 week	4 (25%)
**Description of pain, ***n*** (%)**	
Cold evoked pain	16 (100%)
Burning pain	11 (69%)
Stabbing pain	1 (6%)
Other	4 (25%)
**Baseline pain score (NPRS), Mean (Range; SD), ***n*** (%)**	
Spontaneous pain	6.0 (2.7–8.5; 1.9)
Cold evoked pain	6.8 (5.0 – 8.9; 1.2)
**Medications for pain, n (%)**	
Pregabalin	1 (7%)
Duloxetine	1 (7%)
Amitriptyline	3 (20%)
Gabapentin	2 (13)
Other	2 (13)
**NCS (mean ± SEM; Range)**	
Left sural sensory action potential amplitude	22.2 ± 1.7(13.0–33.0) mV
Left sural nerve conduction velocity	55.9 ± 4.7 (7.7–76.4) m/s
Right sural sensory action potential amplitude	21.1 ± 1.7 (15.0–35.0) mV
Right sural nerve conduction velocity	56.8 ± 2.6 (40.0–72.7) m/s

*SD, Standard Deviation; n, Number of participants; SEM, Standard error of mean; NCS, Nerve conduction studies*.

Participants were given a pain diary to complete, starting on the day of screening and for the next 7 days. The diary collected pain rating scores twice daily. An 11-point Numerical Pain Rating Scale (NPRS), with the 0-anchor point being “no pain”, and the 10-anchor point being “pain as bad as you can imagine”, to describe “pain on average in the last 24 hours”, was used for both spontaneous and evoked pain.

After completing this diary for 7 days, the NPRS score was used to determine eligibility for the study. Only subjects with average pain intensity equal to or greater than 4/10 on the NPRS scale were eligible to participate further in the study (they were unaware of this criterion). They were advised to continue completing the diary daily for the entire duration of the study, until the end-of-study follow up visit.

Application of Capsaicin 8% patch was performed within 1 month from the baseline visit (in 5 subjects a baseline biopsy was available from an earlier time-point, performed for diagnosis with similar findings). 16 participants had follow-up assessments 3 months after Capsaicin 8% patch application.

The mean ± SEM of the Numerical Pain Rating Score (NPRS) at the first visit was 6.0 ± 0.5 and 6.8 ± 0.3 for spontaneous and cold-induced pain respectively (Table 2).

**Table 2:** Results at baseline and at 3 month follow-up for participants with NFCI treated with Capsaicin 8% patch for spontaneous or cold-evoked pain (NPRS; paired *t*-test), Patient Global Impression of Change (PGIC; paired *t*-test, baseline PGIC was change from screening to baseline visit), and the skin biopsies.

**NUMERICAL PAIN RATING SCALE (NPRS) SCORE FOR SPONTANEOUS PAIN [MEAN ± SEM]**						
**Spontaneous pain**	**Baseline**	**3 Month FU**	***p*** **-value**			
	6.0 ± 0.5	4.8 ± 0.6	**p = 0.006			
**Cold evoked pain**	**Baseline**	**3 Month FU**	***p*** **-value**			
	6.8 ± 0.3	5.6± 0.6	**p = 0.006			
	**Baseline**		**3 Month FU**	***p*** **-value**		
**Patient Global Impression of Change (PGIC) [Mean ± SEM]**	4 ± 0.09		3 ± 0.4	***p = 0.0001		
**SKIN BIOPSY**						
			**Mean**	**SD**	**SEM**	***p*** **value;** ***n*** **= 16**
**PGP9.5**	IENF	Baseline	3.36	1.62	0.40	***p < 0.0001
		3 months FU	7.11	2.47	0.62	
			**Mean**	**SD**	**SEM**	***p*** **value;** ***n*** **= 16**
	SENF	Baseline	1.41	0.41	0.10	***p < 0.0001
		3 months FU	2.26	0.64	0.16	
			**Mean**	**SD**	**SEM**	***p*** **value;** ***n*** **= 16**
**GAP43**	SENFs	Baseline	0.24	0.15	0.04	***p = 0.0001
		3 months FU	0.88	0.48	0.12	
			**Mean**	**SD**	**SEM**	***p*** **value;** ***n*** **= 15**
**vWF**		Baseline	3.24	1.25	0.33	*p* = 0.80
		3 months FU	3.35	1.29	0.32	

*SEM, Standard error of the mean; FU, follow up*.

#### Clinical Examination

Clinical examination and tests confirmed that participants had a predominantly sensory neuropathy. Neurological deficits were recorded using the Neuropathy Impairment Score Lower Limbs (NIS-LL) (14), which is a summed score including of muscle power, reflex loss (maximum score 88, indicating severe neuropathy). The mean (SEM) NIS-LL at the baseline visit was 5.7 (1.2).

#### Assessment of Neuropathy

Clinical examination and tests were performed in both lower and upper limbs. Nerve conduction studies were performed by a senior consultant neurophysiologist.

#### Short-Form McGill Pain Questionnaire (SF-MPQ-2)

Symptoms were also recorded using the Short-Form McGill Pain Questionnaire (15), with maximum score 220, indicating severe symptoms. The SF-MPQ-2 is composed of 4 summary scales: (1) continuous, (2) intermittent, (3) neuropathic and (4) affective pain descriptors.

#### Patient Global Impression of Change

Patients were asked to complete the Clinical Global Impression of Change; this was completed at the baseline visit and three-monthly visits. The Questionnaire is composed of a 7-point scale, enabling the patient to indicate no change, improvement or worsening of their condition.

#### Quantitative Sensory Testing

For quantitative sensory testing (QST), thresholds for light touch were measured using Semmes–Weinstein hairs (made by A. Ainsworth, University College London, UK); No. 1 (0.0174 g) to No. 20 (263.0 g). The hair with the lowest force reliably detected by the patient on the dorsum of the toe was recorded. Values >0.0479 g were considered abnormal (16).

Vibration perception thresholds were measured using a biothesiometer (Biomedical Instrument Company, Newbury, OH, USA) placed on the metatarsophalangeal joint of the big toe. Three ascending and three descending trials were carried out, and the mean value obtained. Values >12 V were considered abnormal (17).

Thermal perception thresholds were performed as described (18, 19) using the TSA II–NeuroSensory Analyzer (Medoc, Ramat Yishai, Israel). A 30 × 30 mm thermode was used and thermal thresholds determined in the soles of the feet (under the instep), for warm perception, cool perception, heat pain and cold pain from a baseline temperature of 32°C, with a change in temperature of 1°C/s. The mean of three consecutive tests for each modality was recorded. Mean variations from baseline temperature >6.4°C for warm sensation, >2.3°C for cool sensation and >10.4°C for heat pain, were considered abnormal (20).

#### Nerve Conduction Studies

Nerve conduction and sympathetic skin response (SSR) studies were performed only at baseline to characterise the neuropathy, on a Medtronic Keypoint electromyogram system (Medtronic, Minneapolis, MN, USA) (3). Antidromic sensory action potentials for the sural and superficial peroneal nerves and orthodromic sensory action potentials for the medial and lateral plantar, median, and ulnar nerves on both sides were recorded. For the sural, superficial peroneal, medial plantar, and ulnar nerves, action potentials of <5 μV amplitude were considered abnormal, while for the lateral plantar and median nerves, amplitudes of <2.5 and <10 μV were considered abnormal, respectively. Sensory conduction velocities of <50 and 40 m/s were considered abnormal for upper and lower limb nerves, respectively. The nerve compound action potentials were recorded using standard described techniques, and the issue of skin resistance was mitigated by: i) carrying out pre-recording skin preparation identically in all subjects, and ii) by using the identical type of recording electrodes in all subjects. These were also the methods employed while collecting normative data from our control subjects. SSRs were recorded from both palms and both soles in response to a sudden unexpected tactile stimulus and a sudden inspiratory gasp. An absent SSR was considered abnormal.

#### Calf Skin Biopsy and Immunohistochemistry

3.5-mm diameter skin punch biopsies were collected under local anaesthesia from the distal lateral calf at baseline (Visit 1) before Capsaicin 8% patch application, and repeated 3 months after treatment, within the area of Capsaicin 8% patch treatment. Skin biopsies from age and gender-matched healthy volunteers were analysed alongside as controls.

The immunohistochemical methods and antibodies used were reported in detail previously (3, 19–22). The antibodies were to the pan-neuronal structural nerve marker PGP 9.5 (Rabbit, RA95/06, 1:40,000; Ultraclone, Isle of Wight, UK), the nerve fibre regeneration marker, growth associated protein GAP-43 (G9264, Mouse, 7B10, 1:80,000; Sigma, Poole, UK), and marker for blood vessels von Willebrand factor vWF (Rabbit, 1:10,000; Novocastra Laboratories, Milton Keynes, UK). Briefly, the skin biopsy was immersed in fixative (modified Zamboni's fluid – 2% formalin; 0.01 M phosphate buffer; 15% saturated picric acid (pH 7.2), then washed in phosphate buffered saline (PBS; 0.1 M phosphate; 0.9% w/v saline; pH 7.3) containing 15% w/v sucrose for an hour, before snap freezing in optimum cutting tissue embedding medium (Tissue-Tek OCT, RA Lamb Ltd, Eastbourne, U.K.). Frozen sections were collected for processing (50μm for PGP9.5, 30 μm for GAP-43, and 15 μm for vWF). For GAP-43 and vWF, sections were collected onto poly-L-lysine (Sigma, Poole, UK) coated glass slides; for PGP9.5, 50μm sections were floated onto PBS in 12-well plates, and processed further as described (3). Sites of primary antibody attachment were revealed using nickel-enhanced, avidin-biotin peroxidase (ABC - Vector Laboratories, Peterborough, UK). Sections were counterstained for nuclei in 0.1% w/v aqueous neutral red, air dried and mounted in xylene-based mountant (DPX; BDH/Merck, Poole, UK).

Nerve fibres were counted along the length of four non-consecutive sections. The length of epithelium in each counted section was measured using computerised microscopy software (Olympus ANALYSIS 5.0 Soft, Olympus UK, Southend, Essex, UK) and results expressed as fibres/mm length of the section. Sub-epidermal nerve immunoreactivity was obtained as a percentage (% area), measured by image analysis where digital photomicrographs were captured via video link to an Olympus BX50 microscope. The grey-shade detection threshold was set at a constant level to allow detection of positive immunostaining and the area of highlighted immuno-reactivity was expressed as a percentage (% area) of the field scanned. Images were captured (×40 objective magnification) along the entire length, and the mean values were used for statistical analysis. The GAP43 IENFs were sparse and so only SENFs were analysed.

Skin biopsy analyses and quantification were performed independently of the clinical data collection. Validation of these methods for assessment of skin biopsies have been published previously (22).

### Statistical Analysis

Data were analysed using GraphPad Prism version 5.0 for Windows (GraphPad Prism Software, San Diego, CA, USA). The statistical tests used were paired two-tailed Mann-Whitney test, Student's *t-*test, two-way ANOVA analysis, Dunnett's Multiple Comparison Test, and Spearman's correlation test. For all statistical tests, *p* values < 0.05 were considered significant.

## Results

### Material and Methods

#### Pain Scores and Questionnaires

**Figure 1:**
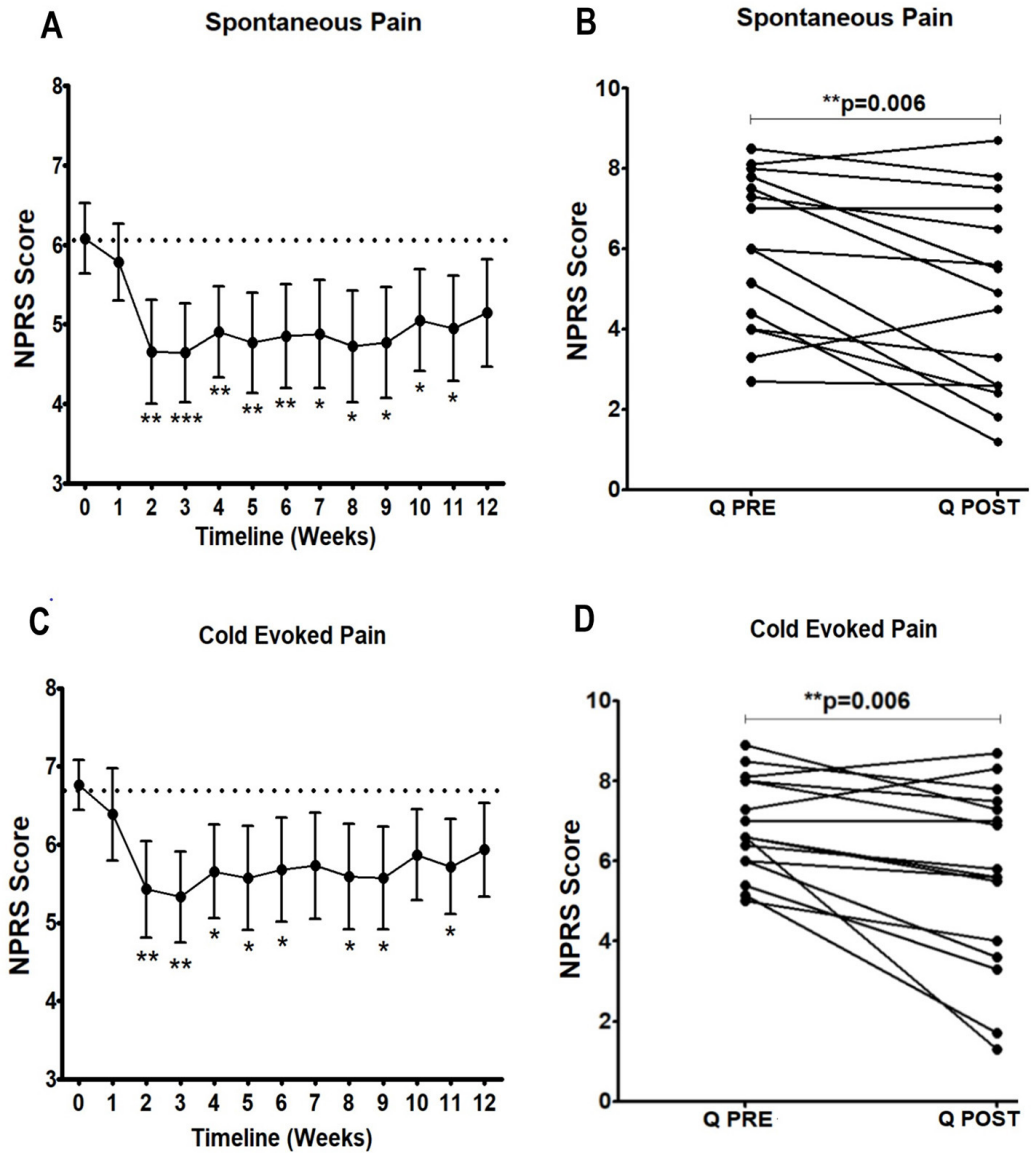
Time-course of pain relief in participants with NFCI treated with Capsaicin 8% patch. Time-course of pain scores for spontaneous pain **(A, B)** and cold evoked pain **(C, D)**. Repeated measures ANOVA for baseline to week 12 period was statistically significant for spontaneous pain (*F* = 2.4; **p = 0.005); and cold evoked pain (*F* = 2.1; *p = 0.01); statistically significant difference was observed for the overall pain scores 3 months after the treatment (paired *t* test, **B** and **D**). 95% CI shown for spontaneous pain weekly **(A, C)**, and *p* values, with Dunnett's Multiple Comparison Test (for a significance level of 0.05).

There was a significant reduction in the NPRS scores three months after Capsaicin 8% patch application (baseline week vs. week 12) for both spontaneous (Table 2, Figures 1A,B) and cold-evoked pain (Figures 1C,D). The mean difference of NPRS score between baseline and 3-month follow-up visit was −1.1 (95% CI: 0.37 to 1.90; ^**^p = 0.006) for spontaneous pain, and −1.2 (95% CI 0.40 to 2.04; ^**^p = 0.006) for cold-evoked pain. Reduction from baseline was 20%, which would generally be regarded as clinically significant.

At follow-up 3 months after the treatment, SF-MPQ-2 did not show significant reduction in overall pain score (*p* = 0.23) or pain scores recorded by using other descriptor category groups (continuous pain, *p* = 0.11; intermittent pain, *p* = 0.32; affective pain, *p* = 0.64; neuropathic pain, *p* = 0.30).

There was no significant difference in the NIS-LL score 3 months after the treatment of capsaicin 8% patch (*p* = 0.78).

The Capsaicin 8% patch application was well-tolerated, there were no adverse events reported.

#### Nerve Conduction Studies

Four participants (25%) showed abnormalities, restricted to the sensory action potential amplitude of plantar nerves. Sural sensory action potential (mean ± SEM) was 22.2 ± 1.7 (13.0–33.0) μV and 21.1 ± 1.7 (15.0 – 35.0) μV in left leg and right leg respectively. The sural nerve velocity (mean ± SEM) was 55.9 ± 4.7 (7.7–76.4) m/s in left leg and 56.8 ± 2.6 (40.0–72.7) m/s in right leg (Table 1). SSRs from the palms and soles were normal in all cases.

#### Quantitative Sensory Testing

There were no significant changes in monofilament (*p* = 0.17), vibration (*p* = 0.45), or thermal thresholds (cool: *p* = 0.21; warm: *p* = 0.18, cold pain: *p* = 0.13; heat pain: *p* = 0.32). There were no baseline QST predictors of higher pain relief response (or baseline predictors of skin biopsy nerve markers).

### Immunohistochemistry

#### PGP9.5 IENFs and SENFs

**Figure 2:**
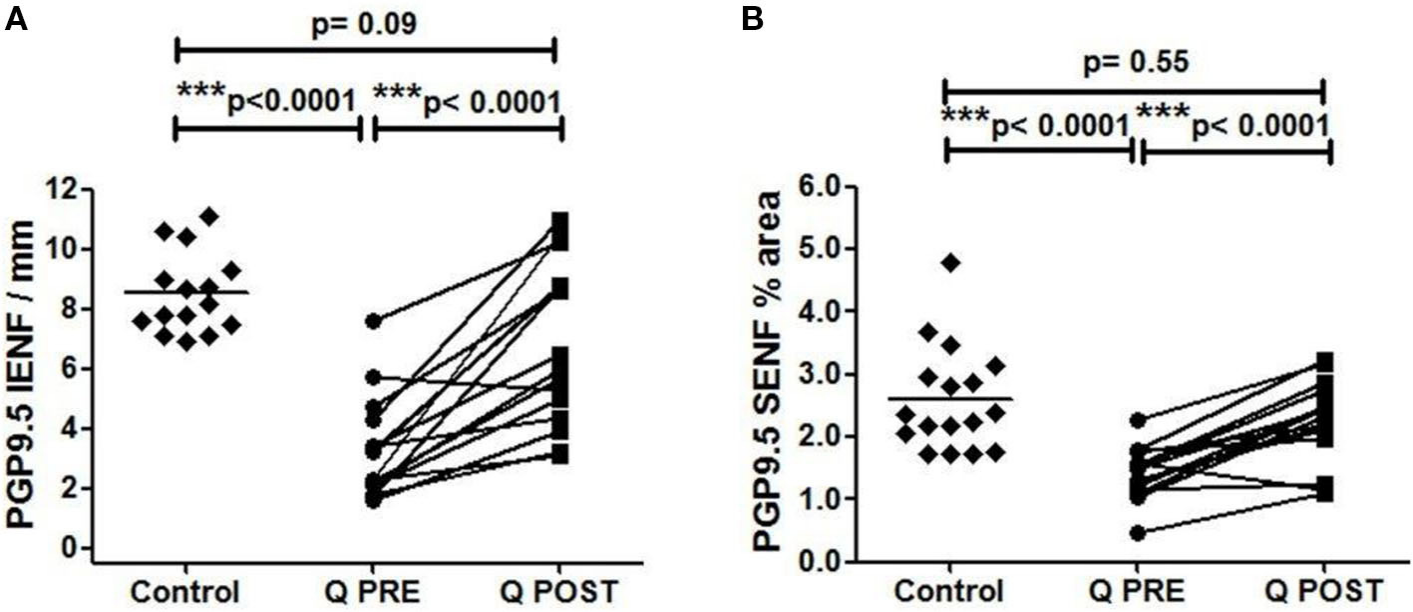
Immunohistochemistry results in skin biopsies for PGP9.5-positive nerve fibres. **(A)** Intra-epidermal PGP9.5 nerve fibres (IENFs/mm) at baseline (Q PRE), and significant increase at 3 months after treatment (Q POST) with Capsaicin 8% patch Qutenza (Q) (paired *t*-test). Statistically significant difference between control group and baseline (Q PRE), but not significant after treatment (Q POST) (Mann-Whitney test). **(B)** Sub-epidermal PGP9.5 nerve fibres (SENFs; % Area) at baseline (Q PRE) and 3 months after treatment (Q POST) with Capsaicin 8% patch Qutenza (Q). Statistically significant increase after Capsaicin 8% patch application (paired *t-*test). Statistically significant difference between control and treated group before (Q PRE) but not after (Q POST) Capsaicin 8% patch application (Mann-Whitney test).

**Figure 3:**
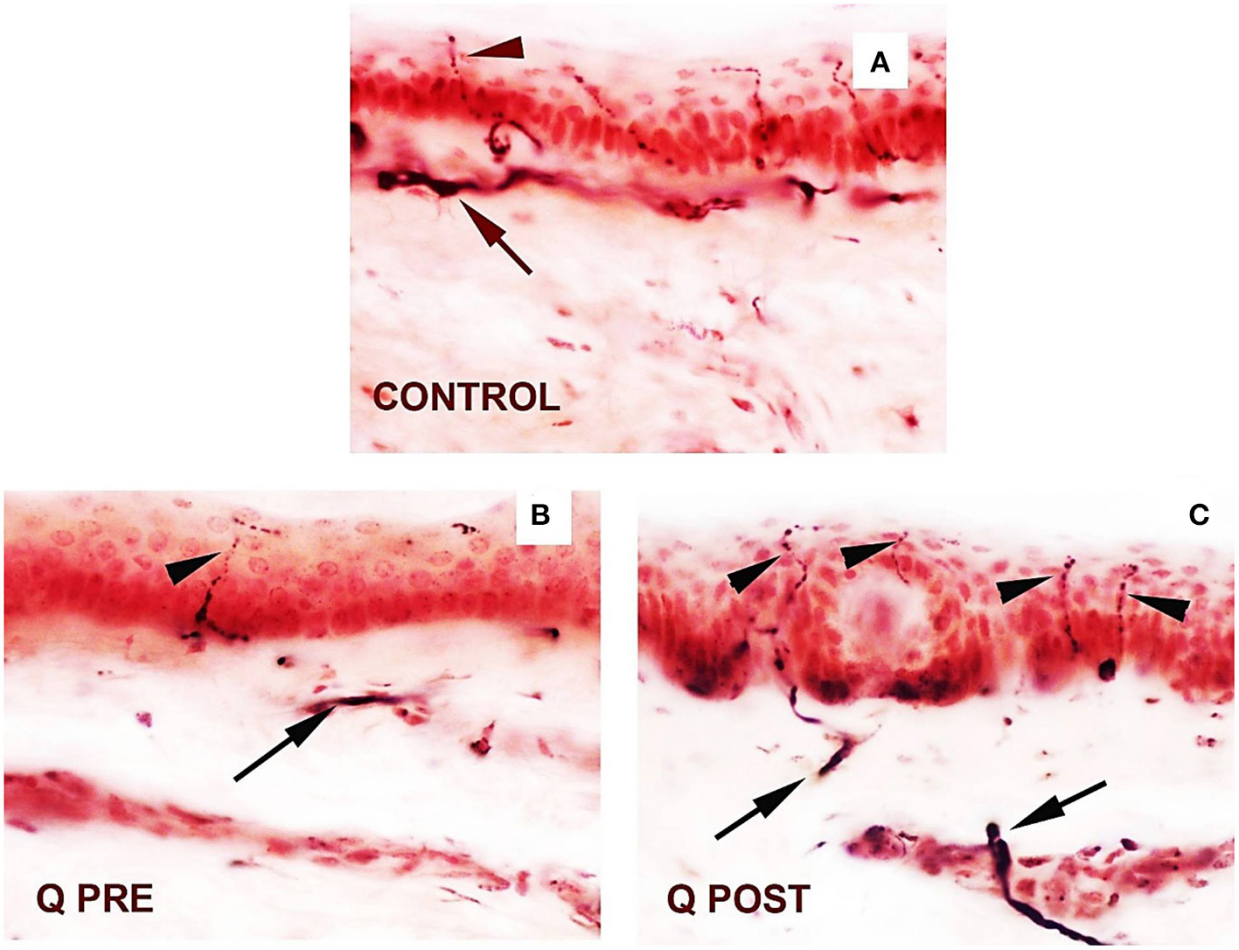
Skin sections staining for PGP9.5 before and after application of Capsaicin 8% patch. **(A)** Control skin biopsy section, from a healthy human volunteer (intra-epidermal nerve fibre marked with arrowhead, and sub-epidermal nerve fibre with arrow). **(B)** Skin biopsy section from a participant with NFCI, pre-treatment (Q PRE); few intra-epidermal nerve fibres and sub-epidermal nerve fibres were observed before Capsaicin 8% patch Qutenza (Q) application. **(C)** Skin biopsy section from same participant as above with NFCI, post-treatment (Q POST); 3 months after Capsaicin 8% patch application, the abundance of both the IENFs and SENFs appeared restored. Magnification × 40.

Calf skin biopsies showed a significant increase in the density of PGP9.5-positive intra-epidermal fibres (IENFs) (^***^p < 0.0001, Table 2, Figures 2A, 3). PGP9.5 IENFs were significantly lower before but not after Capsaicin 8% patch application when compared to healthy controls.

PGP9.5 sub-epidermal fibres (SENFs) were increased significantly after Capsaicin 8% patch application (^***^p < 0.0001, Table 2, Figures 2B, 3). PGP9.5 SENFs were significantly lower before but not after Capsaicin 8% patch application, when compared to healthy controls.

#### GAP43 SENFs

**Figure 4:**
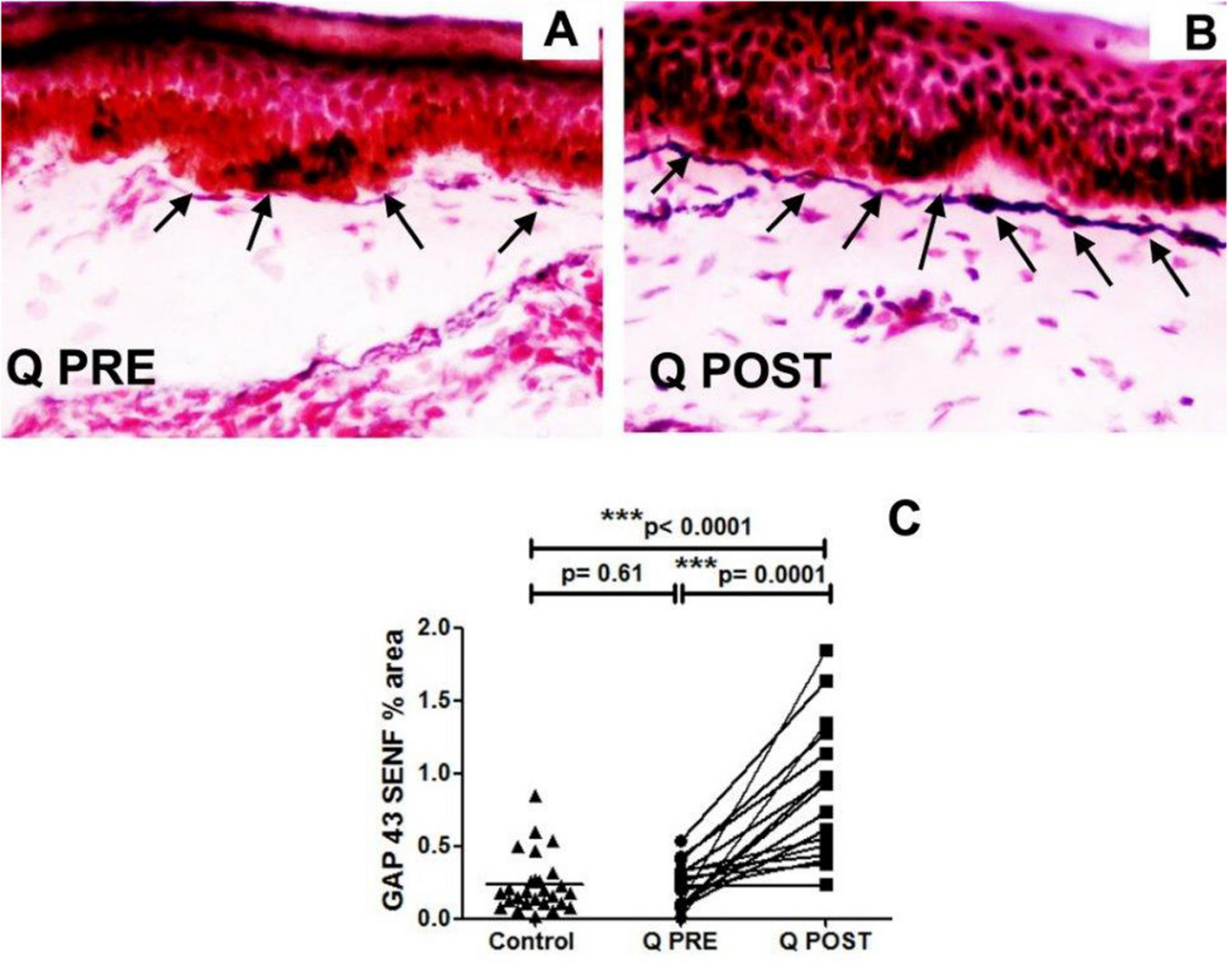
Immunohistochemistry results in skin biopsies for GAP3 nerve fibres. **(A)** NFCI skin biopsy section before treatment with Capsaicin 8% patch (Q PRE): sub-epidermal nerve fibres (arrows) were present, at a density similar to control/normal skin. **(B)** NFCI skin biopsy section in the same participant as above, post-treatment (Q POST); skin biopsy collected 3 months after Capsaicin 8% patch Qutenza (Q) application: note the marked increase of GAP 43 positive SENFs in length and thickness. Original magnification ×20. **(C)** Sub-epidermal GAP43 nerve fibres (SENFs; % Area) at pre-treatment visit (Q PRE) and visit 3 months after treatment (Q POST) with Capsaicin 8% patch Qutenza (Q). Statistically significant increase of SENFs at 3 months after Capsaicin 8% patch application (paired *t-*test). Statistically non-significant difference between control group and treated group before treatment (Q PRE), but statistically significant difference between control group and treated group after Capsaicin 8% patch application (Q POST) (Mann-Whitney test).

These was a significant increase in sub-epidermal nerve fibres positive for GAP43 after treatment (^***^p = 0.0001; Table 2, Figures 4A–C). GAP43 SENFs showed no significant difference from controls before Capsaicin 8% patch application but were significantly increased after Capsaicin 8% patch application (^***^p < 0.0001; Figure 4C).

#### vWF

No changes in von Willebrand Factor (vWF) were observed after treatment (Table 2, note *n* = 15 sample pairs, one sample could not be analysed for this marker for technical reasons). Comparison with controls showed significantly higher levels of vWF both before and after treatment (^**^p < 0.0001 and ^**^p = 0.007, respectively), as we have reported previously in a different cohort of subjects with NFCI (3).

### Correlations

**Figure 5:**
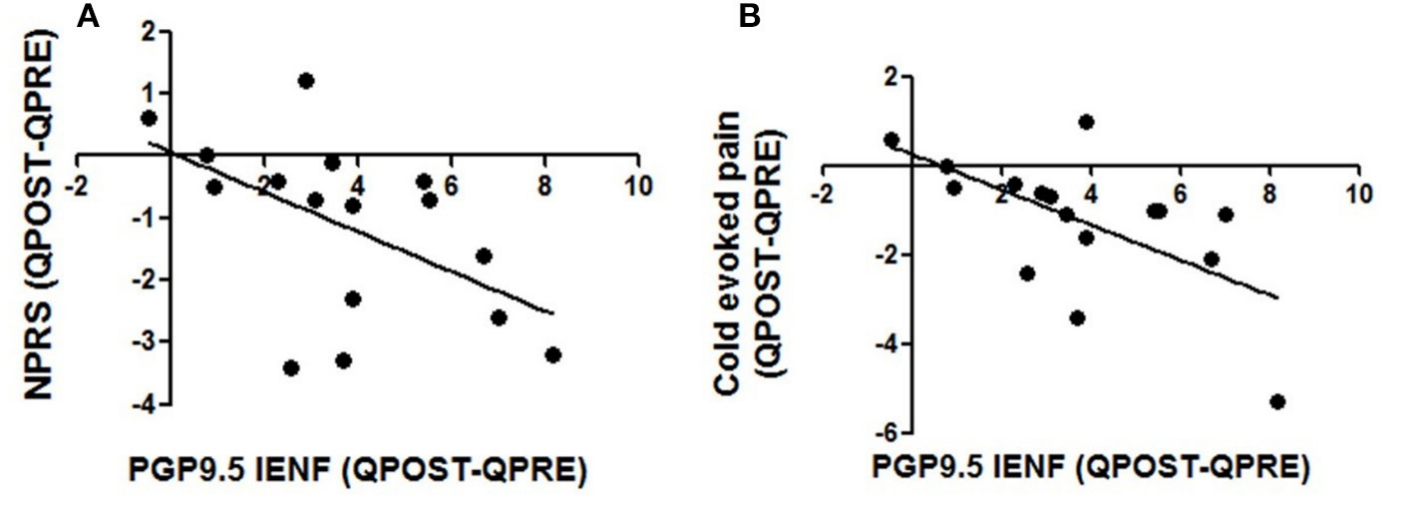
Correlations between changes in intra-epidermal nerve fibre density and pain relief. Decrease in pain scores in spontaneous pain **(A)** and cold evoked pain **(B)** were correlated with changes in density of PGP 9.5 nerve fibres 3 months after the application of Capsaicin 8% patch, Spearman test; p values were respectively *p* = 0.027 and *p* = 0.019.

The increase of IENFs correlated significantly with reduction of spontaneous (Spearman r: −0.55; *p* = 0.027) and cold-evoked pain (Spearman r: −0.57; *p* = 0.019; Figure 5).

## Discussion

NFCI affects civilians exposed to cold environments relating to certain occupations and leisure activities, but especially to military service personnel during training or active duty. While the improvement of preventive measures has helped reduce the incidence of NFCI, there is a major current unmet need for the effective treatment of chronic neuropathic pain in NFCI, and the underlying pathological changes.

In this initial study, the Capsaicin 8% patch (Qutenza) as licenced was well-tolerated and effective for reducing neuropathic pain, for up to 3 months after a single 30-minute application. Participants with chronic NFCI symptoms showed significant decrease in both spontaneous pain and cold-evoked pain, and the time-course of pain relief over 3 months following Capsaicin 8% treatment was virtually identical to other painful neuropathies e.g., painful diabetic neuropathy and chemotherapy induced painful neuropathy (10–13). Importantly, the Patient Global Impression of Change showed significant improvement. NIS-LL score was unchanged, which was not unexpected, as it includes motor function, which is not usually affected in NFCI. Quantitative sensory tests showed no change, as previously (10, 13); it may be early to see functional changes with QST just 3 months after treatment.

The symptoms of NFCI are generally treated with current oral medications used for chronic pain, including neuropathic pain, though some studies have assessed the efficacy of new treatments (23). A range of nerve fibre, vascular and ion channel mechanisms have been reported by us to underlie chronic NFCI (3), which represent potential new targets for pain relief. A key finding is loss of intra-epidermal nerve fibres, which comprise small sensory fibres, including nociceptors. This reduction in intra-epidermal nerve fibre density in the skin, with dysfunction of the residual small sensory nerve fibres e.g., hyperactivity, is present in many other causes of painful neuropathy (3, 10, 13).

The mechanisms of neuropathic pain in these neuropathies, and the effect of Capsaicin 8% patch, has been reviewed in detail previously (3, 10, 19). It has also been shown by us, in another chronic neuropathic condition (chemotherapy-induced painful neuropathy, CIPN), to produce pain relief and regeneration and restoration of sensory nerve fibres i.e. ameliorate the pathological changes (13). Our hypothesis was that a single application of capsaicin 8% patch may relieve pain and also induce nerve regeneration or restoration of the nerve fibre phenotype, by “pruning” the abnormal small sensory fibres (nociceptors), which selectively express TRPV1, the heat and capsaicin receptor. As the patients were now not receiving chemotherapy (and here not re-exposed to Non-freezing cold injury conditions recently), which may adversely affect both nerves and their target skin cells (3, 10, 13), hence nerve fibres may regenerate more “normally”. In accord with our hypothesis, this study showed a significant increase of small sensory nerve fibres (IENFs) with PGP9.5, and also of regenerating nerve fibres with their selective marker GAP43. Further, the increase of IENFs correlated significantly with reduction of spontaneous and cold-evoked pain.

Randomised placebo-controlled clinical trials are clearly required in NFCI, based on our initial findings. Previous studies with the Capsaicin 8% patch in patients with painful diabetic neuropathy provide useful insights into mechanisms and support future clinical trials. In one study, the effects of a single Capsaicin 8% patch application identified subgroups i.e. 32% showed reduction in pain that was maximum in week 3, followed by a slow return towards baseline, 34% showed reduction in pain that persisted at week 12 i.e. no return to baseline pain, and 31% showed no change in pain scores (24). A repeat treatment safety study, with 2-monthly Capsaicin 8% patch over a 12 month period, showed progressive improvement of pain scores in patients with diabetic peripheral neuropathy, compared to standard of care treatment alone (25). These studies support a randomised clinical trial with repeated 3-monthly application of the Capsaicin 8% patch in NFCI subjects over one year, with use of low dose (0.075%) patch as placebo, to study the reversal of pain symptoms and pathological changes in NFCI.

Further studies may elucidate the mechanisms and functional effects of the Capsaicin 8% patch, particularly the time-course in relation to pathophysiological changes that underlie chronic NFCI (3). It is proposed that the milieu for regeneration and phenotypic maturation would be more favourable for the residual small sensory nerve fibres “pruned” by capsaicin in this NFCI study, as previously in CIPN (13). However, this favourable milieu requires molecular characterisation, including changes in expression of cutaneous neurotrophic factors, and their interactions with regenerating nerve fibres. These interactions may lead to normalisation of stimulus transduction at nerve terminals e.g., mediated by TRP receptors, and the onward propagation of impulses e.g., via sodium channels. Sensory receptor and ion channel changes for sensory nerve function may thus take longer than structural nerve improvements i.e. longer than 3 months. Similar regenerative effects may occur following terminal axotomy of nerve fibres not expressing TRPV1, by agents other than capsaicin.

There is no simple correlation between intra-epidermal nerve fibre density and neuropathic pain, or sensory thresholds. Early serial sensory testing after Capsaicin 8% patch treatment e.g., when most effective for pain relief in NFCI, and at later stages after repeat applications, are thus of interest. Capsaicin has significant effects only on TRPV1 expressing cells, the small sensory nerve fibres, and not on blood vessels, hence the latter did not show changes in this study; the vasculature may change over time following interactions with regenerated nerve fibres, and lack of exposure to cold conditions.

In conclusion, this study provides evidence for treatment of NFCI with the Capsaicin 8% patch Qutenza, for both neuropathic relief, and regeneration and restoration of nerve fibres. However, randomised clinical trials are required, to establish its effects on pain relief and “disease-modification” i.e. reversal of the underlying pathophysiological effects.

## Data Availability Statement

The original contributions presented in the study are included in the article/supplementary files, further inquiries can be directed to the corresponding author/s.

## Ethics Statement

The studies involving human participants were reviewed and approved by Ministry of Defence Research Ethics Committee (Ethics reference number: 890/MoDREC/18). The patients/participants provided their written informed consent to participate in this study.

## Author Contributions

PA and DW organised patient referrals and coordination of assessments. RP helped with clinical assessments. VPM performed the nerve conduction studies. PD analysed the skin biopsies. All authors contributed to drafting the manuscript and approved the final manuscript.

## Conflict of Interest

The authors declare that the research was conducted in the absence of any commercial or financial relationships that could be construed as a potential conflict of interest.

## Publisher's Note

All claims expressed in this article are solely those of the authors and do not necessarily represent those of their affiliated organizations, or those of the publisher, the editors and the reviewers. Any product that may be evaluated in this article, or claim that may be made by its manufacturer, is not guaranteed or endorsed by the publisher.
